# Disparities in the timing of antenatal care initiation and associated factors in an ethnically dense maternal cohort with high levels of area deprivation

**DOI:** 10.1186/s12884-022-04984-6

**Published:** 2022-09-19

**Authors:** Shuby Puthussery, Pei-Ching Tseng, Esther Sharma, Angela Harden, Malcolm Griffiths, Jacqueline Bamfo, Leah Li

**Affiliations:** 1 Maternal and Child Health Research Centre, Institute for Health Research, University of Bedfordshire, Park Square, Luton, LU1 3JU Bedfordshire UK; 2 School of Health Sciences, Division of Health Services Research and Management, City, University of London, Northampton Square, EC1V 0HB London, England; 3 Department of Obstetrics and Gynaecology, The Luton and Dunstable University Hospital NHS Foundation Trust, Lewsey Rd, Luton, LU4 0DZ Bedford UK; 4 Population, Policy and Practice Research and Teaching Department, Great Ormond Street Institute of Child Health, University College London, London, WC1N 1EH UK

**Keywords:** Antenatal care, Ethnicity, Maternal health, Diversity, Social disadvantage

## Abstract

**Background:**

Late access to antenatal care is a contributor to excess mortality and morbidity among ethnic minority mothers compared to White British in the UK. While individual ethnicity and socioeconomic disadvantage are linked to late antenatal care initiation, studies have seldom explored patterns of late initiation and associated factors in ethnically dense socially disadvantaged settings. This study investigated disparities in the timing of antenatal care initiation, and associated factors in an ethnically dense socially disadvantaged maternal cohort.

**Methods:**

A retrospective cross-sectional study using routinely collected anonymous data on all births between April 2007—March 2016 in Luton and Dunstable hospital, UK (*N* = 46,307). Late initiation was defined as first antenatal appointment attended at > 12 weeks of gestation and further classified into moderately late (13–19 weeks) and extremely late initiation (≥ 20 weeks). We applied logistic and multinomial models to examine associations of late initiation with maternal and sociodemographic factors.

**Results:**

Overall, one fifth of mothers (20.8%) started antenatal care at > 12 weeks of gestation. Prevalence of late initiation varied across ethnic groups, from 16.3% (White British) to 34.2% (Black African). Late initiation was strongly associated with non-White British ethnicity. Compared to White British mothers, the odds of late initiation and relative risk of extremely late initiation were highest for Black African mothers [adjusted OR = 3.37 (3.05, 3.73) for late initiation and RRR = 4.03 (3.51, 4.64) for extremely late initiation]. The odds did not increase with increasing area deprivation, but the relative risk of moderately late initiation increased in the most deprived ([RRR = 1.53 (1.37, 1.72)] and second most deprived areas [RRR = 1.23 (1.10, 1.38)]. Late initiation was associated with younger mothers and to a lesser extent, older mothers aged > 35 years. Mothers who smoked during pregnancy were at higher odds of late initiation compared to mothers who did not smoke.

**Conclusions:**

There is a need to intensify universal and targeted programmes/services to support mothers in ethnically dense socially disadvantaged areas to start antenatal care on time. Local variations in ethnic diversity and levels of social disadvantage are essential aspects to consider while planning services and programmes to ensure equity in maternity care provision.

## Introduction

Antenatal care, defined as the care provided to an expectant mother by health care professionals from the time the pregnancy is confirmed until the onset of labour, has long been recognised as an effective way of maximising positive birth outcomes for pregnant women and their babies. A number of national and international guidelines have highlighted the importance of timely initiation of antenatal care [1–6]. In the UK, women are recommended to have their first contact with maternity services within the first 10 weeks of pregnancy and subsequently have 7–10 visits for the duration of pregnancy depending on parity and complications [6]. The timely initiation of antenatal care is a key measure of maternity care access in general and has been increasingly linked to birth outcomes including maternal mortality as demonstrated by various epidemiological studies [7–10]. Various factors have been associated with late initiation or inadequate uptake of antenatal care such as ethnicity, socioeconomic status, maternal age, parity, marital status, structural and organisational aspects, lack of transportation, lack of health insurance, failure to recognise pregnancy symptoms and the planned place of delivery [11–16].

Women from Black and minority ethnic backgrounds are found to be at greater risk of initiating antenatal care later than recommended gestational week compared to White women in the UK as elsewhere [12–16]. Systematic reviews that have explored the barriers to and facilitators of early initiation of antenatal care among migrant and ethnic minority women have identified various individual-, family-, social- and health service-related aspects that affected their care pathway [17–19]. These factors included a number of elements such as lack of a fixed abode; preference for local services that are either unavailable and/or inaccessible; lack of joined-up services and difficulty in navigating through the services; inability to access information; perceived impersonal and insensitive nature of the health system; women’s as well as professionals’ lack of knowledge regarding entitlement to care; women’s lack of knowledge about available services, purpose of care and choices available; professionals’ failure to direct women to appropriate care and poor relationships and negative interactions with health professionals; and individual knowledge, culture, motivations and beliefs [17–19].

Late and differential access to antenatal care has also been posited as a plausible explanatory factor for excess mortality and severe morbidity and among mothers from ethnic minority groups compared to White British women in the UK [10]. For example, the recent Confidential Enquiries into Maternal Deaths in the UK [10] found that only 29% of women who died in 2016–18 received the full schedule of antenatal care as set out in the NICE (2017) guidelines [20] and that the risk of maternal death among Black women is four times greater than for white women [10]. While these studies offer relevant insights, they are often limited by smaller samples from ethnic minority populations.

Similar to ethnic minority status, maternal socioeconomic deprivation has frequently been cited as a predictor to delayed or inadequate antenatal care access [21–23]. Feijen-de Jong EI et al. reported living in neighbourhoods with higher rates of unemployment, single parent families, medium-average family incomes, low-educated residents, and women reporting Canadian Aboriginal status as contextual factors for inadequate use of prenatal care or entering care after 6 months [21]. A national survey of 5,332 women living in England found that the most deprived women were 60% less likely to have received any antenatal care, 38% less likely to have been seen by a health professional prior to 12 weeks gestation and 47% less likely to report being able to see a health professional as early as they desired in their pregnancy, when compared to the least deprived women [22]. A study of 10,419 parturient women registered for delivery in four university hospital maternity units in Paris, France, found maternal social deprivation to be independently associated with an increased risk of inadequate antenatal care utilisation [23]. Although the exact mechanisms through which maternal socioeconomic disadvantage impact antenatal care access are not fully understood, evidence has suggested that women’s vulnerability to poor access could be compounded by complex life factors, judgmental and stigmatizing attitudes by health professionals, and differential care provision [24]. A qualitative study on the maternity experiences of mothers with multiple disadvantage in England has reported themes such as ‘a confusing and frightening time’, ‘longing to be respected as an individual’, ‘the importance of choice and control’, and ‘needing trust to feel safe’ reflected in women’s accounts of their care experiences [25].

It has been argued that individuals living in ‘ethnically dense’ areas with members of their own group tend to enjoy better health compared to their counterparts in less ethnically diverse areas [26]. While individual ethnicity, socioeconomic disadvantage, and ethnic density can all be potentially linked to the risk of late initiation of antenatal care, studies have seldom explored the patterns of disparities in the timing of antenatal care initiation and associated factors in settings characterised by high levels of ethnic density and social disadvantage. The aim of this study was to investigate patterns of disparities in late antenatal care initiation, and the associated factors in an ethnically dense socially disadvantaged maternal cohort.

## Methods

We conducted a retrospective cross sectional study using routinely collected anonymous data from Luton and Dunstable Trust hospital, one of the largest National Health Service (NHS) maternity units in the UK. The hospital is situated in Luton, currently ranked the 70^th^ most deprived area from 317 local authorities in England as defined by the nationally derived Indices of Multiple Deprivation (IMD) scores [27, 28]. Luton is ethnically dense with approximately 55% of the population being of Black and Minority Ethnic Origin [29]. The maternity unit delivers approximately 5000 babies per year, providing comprehensive services to women from across a large geographical area. Data relating to all women who give birth in the maternity unit are gathered and stored using a computer based clinical information system, known as the Ciconia Maternity information System (CMiS). We extracted anonymous data from CMiS on all women who have had singleton births between April 2007—March 2016 (*N* = 46,307). Following a preliminary ethical scrutiny by the hospital’s Research and Development Office, a separate ethics approval was deemed to be not required for the study as the study was based on analysis of routinely collected anonymous data. The hospital’s Information Governance Manager ensured adherence to patient confidentiality and data protection before unidentified data were extracted.

### Outcome measures

Data on first antenatal booking appointments that occur in hospital and community practice settings are recorded on CMiS. The system also provides options to record information on ‘booked previously elsewhere’, ‘not formally booked’ and ‘in utero transfer’. The gestational week at first antenatal booking appointment was calculated from the first day of the mother's last menstrual period (self-reported) or based on the dating scan if available, and was available for 45,992 (99.31%) mothers. Consistent with the recommendations from the World Health Organisation [1], late initiation was defined as first antenatal appointment attended at > 12 weeks and further classified into moderately late initiation (13–19 weeks of gestation) and extremely late initiation ≥ 20 weeks of gestation.

### Exposure measures

Maternal ethnicity was self-defined at the mother’s first antenatal visit and was available for 45,799 births, 98.9% of all singletons. For purposes of clarity, the term ‘ethnicity’ as referred in the paper is self-defined and subjective to the person concerned. Ethnic groups in the UK are generally differentiated based on a combination of factors including racial origin, skin colour, cultural and religious affiliation, national and regional origins and language. Details of various ethnic categories, their development and application have been described elsewhere [30]. Ethnicity recording in CMiS is achieved by asking individuals their self-ascribed ethnic category at first antenatal appointment and recording the self-defined ethnicity in accordance with the 2011 UK census categories [30]. We grouped mothers into White British, White Other, Black Caribbean, Black African, Indian, Pakistani, Bangladeshi, and Any other categories. Other exposure measures investigated included maternal age, parity, marital status, maternal smoking during pregnancy, and area level deprivation (based on mothers’ residential postcode). Maternal age was classified into three categories: ‘≤25 years’, ‘26–35 years’ and ‘36 and above years’. Parity was categorised as ‘first’, ‘second’, ‘third’, and ‘fourth or higher order’. Based on the first four characters in the mothers’ address postcode, residential areas were divided into five quintiles from ‘lowest’ to the ‘highest’ based on the IMD scores, with the ‘lowest’ and the ‘highest’ quintile representing the least and most deprived areas respectively. IMD is the official measure of relative deprivation in England and follows an established methodological framework, encompassing a range of individual’s living conditions including income, employment, education, health, crime, barriers to housing and services, and living environment in broadly defining deprivation [27].

### Statistical analysis

Firstly, we applied logistic regression to examine the association of late initiation with each exposure variable, and then with all exposures simultaneously to assess whether they were independently associated with late initiation. We added the year of birth as a covariate in all our analytical models to take into account potential time trends in antenatal care uptake and the demographic composition of the sample. We estimated the unadjusted and adjusted odds ratios (ORs) and 95% confidence intervals (CIs) of late initiation (vs early initiation) for each ethnic group (vs White British) and area deprivation quintile (vs < 20^th^ centile), maternal age ‘ ≤ 25 years’ or ‘36 and above’ (vs 26–35 years), maternal smoking (vs non-smoker), parity (vs first born), and marriage status ‘never married’, ‘divorced/separated/widowed’ (vs married). Secondly, to establish whether associations differed for moderately and extremely late initiation, we applied multinomial logistic regression models and estimated relative risk ratios (RRRs) and 95% CIs for moderately and extreme late initiation (vs early initiation) for each exposure and all exposures simultaneously. To establish the potential interaction between each exposure variable and area deprivation, we examined the association between exposure variables and late initiation of antenatal care stratified by deprivation quintiles. These analyses were conducted using a sample of 44, 809 births with complete data on gestational week at antenatal care initiation, ethnicity, and other exposure variables (97.7% of all singletons).

In addition, to assess whether there were ethnic differences in late initiation given whether the mother was UK-born, we applied logistic regression to late initiation (> 12 weeks) on ethnic groups and migrant status, based on place of birth as self-reported by mothers. This analysis was conducted on 20,100 mothers with information on place of birth. The place of birth information was missing for more than 75% of births up to the year 2013 as it was optional to record this information until then and was mostly recorded for women born in the UK. The place of birth information was available for more than 98% of births from the year 2013 onwards. All analyses were performed using IBM Statistics Package for the Social Sciences (SPSS)® v21.

## Results

**Fig. 1:**
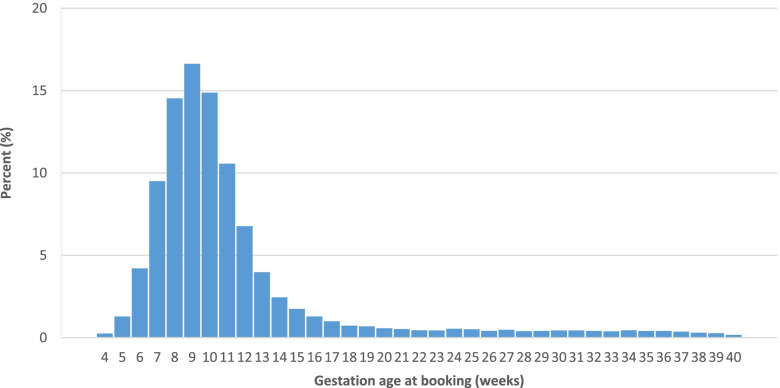
Distribution of gestation age at booking

Of the 46,307 singleton births, gestational week at first antenatal booking appointment was available for 45,992 (99.31%) births among which 79.2%, 12% and 8.8% booked at ≤ 12 weeks, 13–20 weeks and > 20 weeks respectively (Table 1). The distribution of gestational age at booking is displayed in Fig. 1. Maternal country of birth was recorded for 20,100 births with about two fifths (42%) of mothers born abroad (Table 1). About one third (15,916, 34.8%) of all births were to Black Caribbean, Black African, Indian, Pakistani, and Bangladeshi mothers. Maternal characteristics of the total sample by ethnicity were reported [31]. Vast majority (85.8%) lived in neighbourhoods that were in the three most deprived IMD quintiles, and the proportion was higher for mothers from ethnic minority groups (96.1%) with more than half (52.98%) living in the most deprived area quintile [31].

**Table 1:** Maternal characteristics by gestational age at booking [*N* = 45,992]

**Characteristics**	**Total**	** ≤ 12 weeks**	** > 12 weeks**^a^	**13–19 weeks**^b^	**20 weeks and above**^c^
	45,992	36,408 (79.2%)	9584 (20.8%)	5504 (12.0%)	4080 (8.8%)
**Maternal ethnicity**					
White British	20,029(44.0%)	16,758(83.7%)	3271(16.3%)	1834(9.2%)	1437(7.2%)
White Other	6875(15.1%)	4996(72.7%)	1879(27.3%)	946(13.8%)	933(13.6%)
Black Caribbean	880(1.9%)	623(70.8%)	257(29.2%)	164(18.6%)	93(10.6%)
Black African	2490(5.5%)	1638(65.8%)	852(34.2%)	482(19.4%)	370(14.9%)
Indian	1708(3.8%)	1387(81.2%)	321(18.8%)	172(10.1%)	149(8.7%)
Pakistani	7698(16.9%)	6162(80.0%)	1536(20.0%)	1009(13.1%)	527(6.8%)
Bangladeshi	3037(6.7%)	2442(80.4%)	595(19.6%)	434(14.3%)	161(5.3%)
Any other	2772(6.1%)	2048(73.9%)	724(26.1%)	384(13.9%)	340(12.3%)
**Area deprivation quintiles**					
Lowest (least deprived)	4432(9.7%)	3469(78.3%)	963(21.7%)	425(9.6%)	538(12.1%)
Second lowest	1893(4.1%)	1412(74.6%)	481(25.4%)	146(7.7%)	335(17.7%)
Middle	7759(16.9%)	6395(82.4%)	1364(17.6%)	716(9.2%)	648(8.4%)
Second highest	15,993(34.9%)	12,893(80.6%)	3100(19.4%)	1936(12.1%)	1164(7.3%)
Highest (most deprived)	15,993(34.3%)	12,133(77.2%)	3585(22.8%)	2256(14.4%)	1329(8.5%)
**Maternal age (years)**					
≤ 25	13,574(29.5%)	10,243(75.5%)	3331(24.5%)	1879(13.8%)	1452(10.7%)
26–35	26,721(58.1%)	21,686(81.2%)	5035(18.8%)	2918(10.9%)	2117(7.9%)
36 and above	5696(12.4%)	4478(78.6%)	1218(21.4%)	707(12.4%)	511(9.0%)
**Maternal smoking in pregnancy**					
Non-smokers	37,057(81.4%)	29,548(79.7%)	7509(20.3%)	4338(11.7%)	3171(8.6%)
Smokers	8447(18.6%)	6570(77.8%)	1877(22.2%)	1134(13.4%)	743(8.8%)
**Parity**					
First born	18,551(40.3%)	14,342(77.3%)	4209(22.7%)	2025(10.9%)	2184(11.8%)
Second	15,505(33.7%)	12,820(82.7%)	2685(17.3%)	1635(10.5%)	1050(6.8%)
Third	7194(15.6%)	5812(80.8%)	1382(19.2%)	969(13.5%)	413(5.7%)
Fourth or higher	4742(10.3%)	3434(72.4%)	1308(27.6%)	875(18.5%)	433(9.1%)
**Marriage status**					
Married/civil partners	26,465(57.5%)	21,272(58.4%)	5193(54.2%)	3048(55.4%)	2145(52.6%)
Never married	3229(7.0%)	2154(5.9%)	1075(11.2%)	648(11.8%)	427(10.5%)
Divorced/separated/widowed	5788(12.6%)	4548(12.5%)	1240(12.9%)	715(13.0%)	525(12.9%)
Unknown	10,510(22.9%)	8434(23.2%)	2076(21.7%)	1093(19.8%)	983(24.1%)
**Migrant status**					
UK born	8996(58.5%)	7715(61.2%)	1281(46.1%)	741(46.3%)	540(45.7%)
Non-UK born	6392(41.5%)	4892(38.8%)	1500(53.9%)	858(53.7%)	642(54.3%)

^a^Any late antenatal care initiation

^b^Moderately late antenatal care initiation

^c^Extremely late antenatal care initiation

### Maternal characteristics by timing of antenatal care initiation

Table 1 shows maternal characteristics by the gestational week at first antenatal appointment. The prevalence of late initiation varied across ethnic groups, from 16.3% (White British) to 34.2% (Black African). Black African mothers had the highest proportions of late initiation, followed by Black Caribbean (29.2%) mothers whereas mothers from various South Asian groups had similar proportions (18.8%-20.0%). Black African mothers had the highest proportions of extremely late initiation (14.9%) and moderately late initiation (19.4%), more than twice as high as White British mothers (7.2% and 9.25% respectively). Bangladeshi mothers had the lowest proportions of extremely late initiation (5.3%). Overall more than one third (37%) of mothers who booked late were from ethnic minority groups.

Proportions of late bookers were higher (24.5%) among young (≤ 25 years) and older mothers (≥ 36 years) (21.4%), compared to mothers aged 26–35 years (18.8%) at childbirth. Mothers with fourth or higher order pregnancy had highest proportions of late initiation (27.6%), followed by primiparous (pregnant for the first time) mothers (22.7%). Mothers who smoked during pregnancy were more likely to start antenatal care later than non-smokers (22.2% vs 20.3%). Areas in the second lowest deprived IMD quintile had the highest proportions (25.4%) of late booking followed by areas in the most deprived IMD quintile (21.7%) (Table 1).

### Maternal factors associated with late antenatal care initiation

Late initiation of antenatal care was associated with non-White British ethnicity and the association was stronger for Black mothers than South Asian mothers. Compared to White British mothers, the odds of late initiation was greatest for Black African mothers [OR = 2.70 (2.46, 2.96)] and increased after adjusting for other factors [adjusted OR = 3.37 (3.05, 3.73)]. For extremely late initiation (20 + weeks), the relative risk was also highest for Black African mothers [RRR = 2.66 (2.34, 3.02] and increased to fourfold after the adjustment [adjusted RRR = 4.03 (3.51, 4.64]. Black Caribbean mothers were more likely to start antenatal care late [OR = 2.16 (1.86, 2.52)] and the increased odds persisted after the adjustment. For South Asian mothers including Indian, Pakistani and Bangladeshi, the excess odds of late initiation (vs White British mothers) ranged from 24% among Indian mothers [OR = 1.24 (1.09, 1.41)] to 32% among Pakistani mothers [OR = 1.32 (1.24, 1.42)]. The excess odds doubled among Pakistani mothers [adjusted OR = 1.59 (1.47, 1.73)] and Indian mothers [adjusted OR = 1.69 (1.47, 1.93)]. For extremely late initiation, the relative risk was lower among Bangladeshi mothers compared to White British mothers [RRR = 0.78 (0.65, 0.92)]. However, the relative risk increased and was borderline significant after adjustment [adjusted RRR = 1.20 (0.99, 1.44)]. For White Other group, the odds of late initiation was also higher [OR = 2.07 (1.93, 2.21)] and increased after the adjustment [adjusted OR = 2.31 (2.16, 2.48)]. For mothers with data on country of birth, the strength of the association for all ethnic groups weakened after adjusting for migrant status, except for Indian and Pakistani mothers where there was a small increase in the odds of late initiation (Table 2).

**Table 2:** Associations between late antenatal care initiation (> 12 gestational weeks) and exposure variables

	Univariate* (*n* = 44,809)			Model 1** (*n* = 44,809)			Model 2** (*n* = 20,100)
	**OR (95% CI) > 12w**	**RRR (95% CI) 13-19w (5368)**	**RRR (95% CI) 20w + (3848)**	**OR (95% CI) > 12w**	**RRR (95% CI) 13-19w (5368)**	**RRR (95% CI) 20w + (3848)**	**OR (95% CI) > 12w**
**Ethnicity** (baseline** = **W British**)**							
W Other	2.07 (1.93, 2.21)	1.92(1.76,2.09)	2.24(2.05,2.46)	2.31(2.16,2.48)	2.07(1.89, 2.26)	2.65(2.40, 2.92)	1.68(1.42, 1.98)
B Caribbean	2.16 (1.86, 2.52	2.44(2.04,2.93)	1.79(1.43,2.25)	2.37(2.03,2.78)	2.33(1.93, 2.80)	2.41(1.90, 3.04)	1.81(1.32, 2.49)
B African	2.70(2.46,2.96)	2.73(2.43,3.05)	2.66(2.34,3.02)	3.37(3.05,3.73)	2.97(2.63, 3.36)	4.03(3.51, 4.64)	2.45(1.98, 3.03)
Indian	1.24(1.09,1.41)	1.21(1.02,1.42)	1.29(1.08,1.55)	1.69(1.47,1.93)	1.55(1.31, 1.85)	1.89(1.56, 2.29)	1.27(0.98, 1.66)
Pakistani	1.32(1.24,1.42)	1.54(1.42,1.67)	1.04(0.93,1.15)	1.59(1.47,1.73)	1.59(1.43, 1.76)	1.57(1.38, 1.79)	1.28(1.08, 1.52)
Bangladeshi	1.27(1.15,1.41)	1.65(1.48,1.85)	0.78(0.65,0.92)	1.56(1.40,1.75)	1.73(1.52, 1.97)	1.20(1.00, 1.45)	1.21(0.98, 1.50)
Other	1.86(1.69,2.05)	1.78(1.58,2.01)	1.96(1.72,2.23)	2.15(1.95,2.37)	1.88(1.66, 2.13)	2.55(2.22, 2.93)	1.68(1.39, 2.03)
**Area deprivation quintiles** (baseline: < 20^th^ centile, least deprived)							
Second lowest (20-40^th^)	1.26(1.11,1.43)	0.88(0.72,1.08)	1.55(1.33,1.80)	1.14(1.00,1.30)	0.79(0.65,0.97)	1.43(1.22,1.67)	
Middle (40-60^th^)	0.77(0.70,0.85)	0.92(0.81,1.05)	0.65(0.57,0.74)	0.64(0.58,0.71)	0.75(0.66,0.86)	0.55(0.48,0.63)	
Second highest (60-80^th^)	0.87(0.80,0.95)	1.23(1.10,1.38)	0.58(0.52,0.65)	0.60(0.55,0.66)	0.84(0.75,0.95)	0.41(0.36,0.46)	
Most deprived (> 80^th^ centile)	1.07(0.99,1.17)	1.53(1.37,1.72)	0.71(0.63,0.79)	0.68(0.62,0.74)	0.96(0.85,1.08)	0.45(0.40,0.51)	
**Maternal age** (baseline: 26–35 years)							
≤ 25 years	1.35(1.28,1.42)	1.29(1.21,1.38)	1.43(1.33,1.54)	1.42(1.35,1.51)	1.41(1.31,1.51)	1.46(1.35,1.59)	
36 and above	1.19(1.11,1.28)	1.18(1.08,1.30)	1.20(1.08,1.33)	1.07(0.99,1.15)	1.07(0.98,1.18)	1.08(0.97,1.20)	
**Maternal smoking** (baseline: non-smoking)	1.10(1.04,1.17)	1.14(1.06,1.23)	1.04(0.96,1.14)	1.09(1.03,1.17)	1.17(1.08,1.27)	1.00(0.91,1.09)	
**Parity** (baseline: first born)							
Second	0.73(0.69,0.78)	0.92(0.85,0.98)	0.56(0.51,0.60)	0.79(0.75,0.84)	1.00(0.93,1.07)	0.59(0.55,0.64)	
Third	0.85(0.79,0.91)	1.21(1.11,1.32)	0.49(0.44,0.55)	0.96(0.89,1.03)	1.34(1.22,1.46)	0.58(0.51,0.65)	
Fourth or higher	1.36(1.27,1.47)	1.87(1.71,2.05)	0.87(0.78,0.98)	1.65(1.52,1.79)	2.13(1.93,2.35)	1.16(1.03,1.31)	
**Marriage status (**baseline: married/civil partners)							
Never married	1.69(1.55,1.84)	1.58(1.42,1.75)	1.88(1.66,2.14)	1.75(1.60,1.93)	1.77(1.58,1.99)	1.74(1.52,2.00)	
Divorced/separated/widowed	1.20(1.11,1.28)	1.23(1.12,1.34)	1.15(1.04,1.28)	1.26(1.16,1.37)	1.37(1.24,1.51)	1.12(0.99,1.25)	
Unknown	0.99(0.93,1.05)	0.95(0.88,1.03)	1.05(0.97,1.15)	1.01(0.95,1.08)	1.03(0.94,1.11)	1.00(0.91,1.09)	
**Migrant status (non-UK born)**							1.65 (1.46,1.87)

*Unadjusted odds ratios (OR, 95% CI) of late antenatal booking and relative risk ratio (RRR, 95% CI) of moderately and extreme late booking for each potential exposure variable. Unadjusted models included delivery year

**Adjusted models. Model 1 included delivery year and all the exposure variables considered (n= 44, 809). Model 2 included all variables in Model 1, plus mothers’ migrant status (n=20,100)

With respect to area deprivation, the odds of late initiation did not increase with increasing deprivation quintile. However, the relative risk of moderately late initiation increased in the most [RRR = 1.53 (1.37, 1.72)] and the second most deprived areas [RRR = 1.23 (1.11,1.39)], compared with the least derived area. The association disappeared after adjusting for other factors. On the contrary, the relative risk of extreme late initiation was lower in the top two deprived quintiles both before and after the adjustment (Table 2).

Late initiation was also associated with younger mothers [OR = 1.35 (1.28, 1.42)] and to a lesser extent, older mothers (> 35 years) [OR = 1.19 (1.11, 1.28)] compared to those aged 26–35 years. The odds changed little after adjustment for younger mothers, but for older mothers the odds were no longer significant after adjustment.

Mothers who smoked during pregnancy was more likely to start antenatal care late [OR = 1.10 (1.04, 1.17) and it changed little after the adjustment. For extreme late initiation, the relative risk did not differ between smokers and non-smokers. Compared to primiparous women, mothers with one or two previous live births were less likely to book late [OR = 0.73 (0.69, 0.77) and 0.85 (0.79, 0.91) respectively], but the association was no longer significant after the adjustment. Women with three or more previous births were more likely to have late initiation [OR = 1.36 (1.27, 1.47)] and the association persisted after the adjustment. For extremely late initiation, the relative risk was lower among mothers with three or more previous births [RRR = 0.87 (0.78, 0.98)], but it increased after the adjustment [adjusted RRR = 1.16 (1.03, 1.31)]. Mothers who were never married had higher odds of late initiation [OR = 1.69 (1.55, 1.84)] compared to married mothers and the odds ratio changed little after the adjustment. Mothers who were divorced/separated had a modest increase in odds of late initiation [OR = 1.20 (1.11, 1.28)]. Among mothers with information on country of birth data, those who were born aboard were at 65% higher odds of late initiation compared to mothers born in the UK (Table 2). Analyses stratified by deprivation quintiles showed that for most exposure variables, the patterns were similar across the IMD quintiles, except for maternal smoking, which was associated with later booking only among mothers in the three most deprived area quintiles (OR = 1.15, *p* < 0.05).

## Discussion

In our cohort of mothers residing in an ethnically dense area with high levels of deprivation, 79% of women booked antenatal care before 12 weeks of gestation, lower than the national average of 82% [32]. Previous studies have shown that a complex ‘web’ of events and factors influence timely initiation of antenatal care, including pregnancy recognition, cultural understandings of pregnancy as a normal life event that does not warrant care-seeking and access to maternity services [21, 33–37]. Individual-, familial, social- and health service-related aspects such as lack of a fixed abode; preference for local services that are either unavailable and/or inaccessible; lack of joined-up services and difficulty in navigating through the services; inability to access information; perceived impersonal and insensitive nature of the health system; women's as well as professionals’ lack of knowledge regarding entitlement to care; women’s lack of knowledge about available services, purpose of care and choices available; professionals’ failure to direct women to appropriate care and poor relationships and negative interactions with health professionals; and individual knowledge, culture, motivations and beliefs have been shown to affect ethnic minority women’s access to antenatal care in the UK and other high income countries in Europe [17–19]. The higher prevalence of late antenatal care initiation in this cohort may reflect a high incidence of these factors within the pregnant population of the region as a whole. While maternal socioeconomic deprivation is found to be a predictor to delayed or inadequate antenatal care access [21–23], more than two-thirds of the mothers in our cohort lived in areas that were in the two most deprived area quintiles (the most deprived 40% nationwide). It has been suggested that socioeconomically disadvantaged women’s vulnerability to poor access in high income countries could be compounded by complex life factors, judgmental and stigmatizing attitudes by health professionals, and differential care provision [24]. The need to provide accessible and empowering information and guidance to enable these mothers to effectively navigate the system has also been highlighted [25].

Consistent with previous studies [12–16], our analysis showed that timing of antenatal care initiation varied according to maternal ethnicity. More than one third of mothers who had had their initial booking appointment later than 12 weeks in our cohort were mothers from one of the ethnic minority groups, with proportions of late bookers much higher among mothers from Black African and Black Caribbean backgrounds. Our findings also showed that non-White British ethnicity was strongly associated with late or extremely late initiation of antenatal care irrespective of level of area deprivation and Black African and Black Caribbean women were at highest risk. This is consistent with findings from old and new studies that have reported maternal ethnicity as a predictor of late antenatal care initiation [13, 15, 38]. Analyses of national data have shown that Black women and those whose ethnicity was recorded as ‘Other’ were most likely to have their booking appointment after 10 weeks, with 61.5% of Black women and 58.6% women from ‘Other’ ethnicity backgrounds booking later than 10 weeks of pregnancy [39]. Creswell et al. (2013) found that among UK-born women who spoke English in an ethnically diverse urban cohort in the UK, women who identified as from African/Caribbean backgrounds were the only ethnic group at increased risk of late booking compared to White British women [15]. In our cohort, South Asian mothers including Indian, Pakistani and Bangladeshi, were also more likely to start antenatal care late whereas nationally it has been reported that women of mixed, Asian or Chinese ethnicity were least likely to book late for antenatal care, although nearly half of the women from these backgrounds did not book within 10 completed weeks of pregnancy [39]. In our cohort, the effect of ethnicity remained with adjustment for maternal factors including area deprivation, and in most cases showed greater strength of effect on adjustment to other factors, notably with respect to extremely late initiation among Black African mothers. These findings are extremely concerning given that African and Caribbean women have been identified as groups at increased risk of poor maternal and infant outcomes including mortality and morbidity [10, 31]. Some of the known biological risk factors tend to be increasingly prevalent among some ethnic minority groups such as sickle cell anaemia among women of African descent [40] and gestational diabetes among women of South Asian descent [41]. Timely initiation of antenatal care is extremely important to enable healthcare professionals to identify and address any such risk factors and to monitor the health of women and their babies throughout pregnancy.

Our findings indicated that migrant status could be a contributor to late antenatal care initiation for all ethnic groups except for Indian and Pakistani mothers. Inherent to women’s ability to access maternity services is not only the availability of care but also its acceptability and other associated factors including language barriers, cultural sensitivity, and health literacy which can all impact upon migrant women’s access to maternity services in particular [42, 43]. Studies have reported non-English speakers’ difficulties in navigating health services, including confusions regarding entitlement to NHS services, and/or fear of deportation while engaging with health services among some categories of migrants [44, 45]. Recent reports have highlighted the need for healthcare professionals to help and support uptake of antenatal care services by pregnant women who are recent migrants, asylum seekers or refugees, or who have difficulty reading or speaking English, using a variety of means to communicate with women; telling women about antenatal care services and how to use them; undertaking training in the specific needs of women in these groups [10].

Nationally, women from deprived areas were more likely to book later than those from the least deprived populations [22]. In our maternal cohort, the overall risk of late initiation did not increase with increasing area deprivation except for the risk of moderately late initiation in the most deprived and the second most deprived areas compared with the least derived area although the increased risk disappeared on adjustment for other factors. On the contrary, the risk of extreme late initiation was lower in the most deprived and second most deprived areas compared to the least deprived both in the unadjusted and adjusted analyses. Given the equal availability of services in the area, these differences may be attributed to small numbers of mothers living in less deprived areas or to the effect of other unadjusted individual socioeconomic confounders as area deprivation based on the IMD scores reflect a relative rather than an absolute measure [27].

Other factors associated with late antenatal booking in our cohort were mothers being younger (≤ 25 years) or older (> 35 years), as well as being primiparous, possibly because women with no experience of pregnancy were less confident to navigate maternity services or may be unaware about the need to initiate antenatal care early in pregnancy. While older women may feel more confident to navigate the services, they may not feel the need to initiate antenatal care early in pregnancy. Unfavourable previous experiences with maternity or health services in general could also deter women from accessing antenatal services. Nationally, higher proportions of pregnant women aged < 25 years attended the booking appointment at a later stage than older women [39]. In our cohort, mothers who smoked during pregnancy were more likely to start antenatal care late compared to mothers who did not smoke. This is consistent with findings from previous studies that, despite equal availability of antenatal care, women who smoked during pregnancy were likely to start antenatal care later and had fewer contacts with their midwives, compared to women who did not smoke during pregnancy [16, 21]. The link between behavioural factors such as smoking during pregnancy with delayed initiation of antenatal care points to the possible cumulative risk faced by some mothers. Our stratified analysis also suggests potential interactions between area deprivation and smoking in the three most deprived areas where most ethnic minority mothers lived.

This is one of the few studies that used a large recent data set to examine predictors of antenatal care initiation in an ethnically dense area with high levels of deprivation. Our cohort had substantially higher proportions (34.8%) of mothers from ethnic minority groups compared with the maternal population in England and Wales (12.9%) allowing greater statistical power to explore differences by ethnic group. The ability to analyse data at a local level providing opportunities to inform local policy and practice was also a strength of this study. Missing data on country of birth limited the ability to understand the predictors of late initiation of antenatal care at a more granular level, however. Data regarding languages spoken, education levels, father’s country of birth and migrant status would have allowed a greater depth of analysis when looking at factors associated with initiation of antenatal care among this cohort in order to inform the development of targeted interventions. The cross-sectional nature of the study may have limited causal inferences. Few ethnic minority mothers in our sample were from less deprived areas and this affected our ability to compare ethnic differences by the level of area deprivation.

## Conclusion

Compared to the national average, a high prevalence of late antenatal care initiation in our maternal cohort from an ethnically dense area with high levels of social disadvantage has implications for maternity care policy and practice locally, nationally and internationally. With increasing global migration, ethnically dense communities in deprived neighbourhoods are becoming commonplace in many developed countries. Mothers in these communities could potentially be at higher risk of falling behind in terms of their access to maternity services compared with the nation as a whole. The prevalence of late initiation was higher among minority groups including other White, and was particularly high among Black African and Caribbean mothers. The findings also highlight the differences in health care seeking behaviours and the relative inequity in health care access that tend to exist in countries such as the UK where healthcare is considered to be of universal access, accessed free of cost at the point of delivery.

There has been a drive to increase the numbers of women initiating antenatal care in the first 10 weeks of pregnancy through initiatives such as direct self-referrals to local midwifery services in the UK [6]. While these may have resulted in greater timely initiation of antenatal care, our findings highlighted the need to intensify universal and targeted programmes and services to identify and support mothers from ethnic minority groups in areas with high levels of social deprivation who are at greater risk of late antenatal care initiation. The ethnic diversity and levels of social disadvantage should be taken into consideration while planning services and programmes to ensure equity in antenatal care provision.

Future research should include individual indicators of SES in addition to area deprivation to capture the impact of both individual-level and area-level SES on variations in antenatal care initiation. Development and evaluation of effective interventions, co-produced with women, families and maternity care professionals, taking into account cultural beliefs and practices to encourage and support women from ethnic minority groups to engage with maternity services is another area for future research. 

## Data Availability

The datasets generated and/or analysed during the current study are not publicly available due the nature of the agreement with the data provider. The corresponding author will be able to answer any queries related to the data set.

## Abbreviations

NICE: National Institute for Health and Care Excellence
NHS: National Health Service
CMiS: Ciconia Maternity information System
IMD: Indices of Multiple Deprivation
SES: Socioeconomic status
